# Comparison of analgesic effectiveness between nefopam and propacetamol in living kidney donors following rectus sheath block after hand-assisted living donor nephrectomy: a prospective, randomized controlled trial

**DOI:** 10.1186/s12871-024-02607-5

**Published:** 2024-07-02

**Authors:** Won-Jung Hwang, Jung Min Koo, A Rim Yang, Yong Hyun Park, Min Suk Chae

**Affiliations:** 1 Department of Anesthesiology and Pain Medicine, Seoul St. Mary’s Hospital, College of Medicine, The Catholic University of Korea, 222, Banpo-daero, Seocho-gu, Seoul, 06591 Republic of Korea; 2 Department of Anesthesiology and Pain Medicine, Uijeongbu St. Mary’s Hospital, College of Medicine, The Catholic University of Korea, Seoul, Republic of Korea; 3 Department of Urology, Seoul St. Mary’s Hospital, College of Medicine, The Catholic University of Korea, Seoul, Republic of Korea

**Keywords:** Nefopam, Propacetamol, Rectus sheath block, Living donor nephrectomy

## Abstract

**Background:**

Nefopam and propacetamol are the most commonly used analgesics in postoperative multimodal analgesic regimens. Distinct mechanisms are involved in each drug’s anti-nociceptive effects. No studies have compared pain relief efficacy between the two drugs in patients undergoing transplantation surgery. Here, we investigated whether the administration of nefopam or propacetamol to healthy living kidney donors who underwent rectus sheath block (RSB) for parietal pain could reduce the subsequent opioid dose necessary to produce adequate analgesia.

**Methods:**

This prospective, randomized controlled trial included 72 donors undergoing elective hand-assisted living donor nephrectomy into two groups: propacetamol (*n* = 36) and nefopam (*n* = 36). Intraoperative RSB was performed in all enrolled donors. The primary outcome was the total volume of intravenous opioid-based patient-controlled analgesia (PCA) used on postoperative day 1 (POD 1). Additionally, the Numeric Rating Scale scores for flank (visceral) and umbilicus (parietal) pain at rest and during coughing were compared, and the Korean adaptation of the Quality of Recovery-15 Questionnaire (QoR-15 K) was evaluated on POD 1.

**Results:**

Both groups had similar preoperative and intraoperative characteristics. On POD 1, the total amount of PCA infusion was significantly lower in the nefopam group than in the propacetamol group (44.5 ± 19.3 mL vs. 70.2 ± 29.0 mL; *p* < 0.001). This group also reported lower pain scores at the flank and umbilical sites and required fewer rescue doses of fentanyl in the post-anesthesia care unit. However, pain scores and fentanyl consumption in the ward were comparable between groups. The QoR-15 K scores were similar between groups; there were substantial improvements in breathing, pain severity, and anxiety/depression levels in the nefopam group. The incidences of postoperative complications, including sweating and tachycardia, were similar between groups.

**Conclusion:**

Compared with propacetamol, nefopam provides a greater analgesic effect for visceral pain and enhances the effects of blocks that reduce the opioid requirement in living kidney donors with parietal pain managed by RSB.

**Trial registration:**

The trial was registered prior to patient enrollment in the clinical trial database using the Clinical Research Information Service (registration no. KCT0007351, Date of registration 03/06/2022).

## Background

Kidney transplantation significantly improves the quality of life and reduces mortality for patients with end-stage renal disease [1]. Notably, kidneys from living donors result in better patient and graft survival rates than those from deceased donors, underscoring the importance of altruistic living donors [2]. The hand-assisted laparoscopic donor nephrectomy (HALDN) technique is advantageous over conventional methods by reducing analgesic need, hospital stay, and recovery time. Additionally, it manages ureteral and vascular complications more effectively [3]. However, HALDN is still associated with postoperative pain, mainly due to the large supraumbilical incision. This pain includes parietal and visceral components, as well as shoulder-referred discomfort [4].

Managing postoperative pain in living donors, who are typically healthy and pain-free before surgery, remains challenging despite surgical advancements and improved psychological support [5, 6]. Effective pain management must address both parietal and visceral pain resulting from surgical incisions and retractions [7, 8]. While intrathecal morphine can reduce both types of pain, it carries risks like cerebrospinal fluid leakage [9, 10]. Fascial plane blocks are beneficial for parietal pain but may not significantly reduce overall postoperative pain in living donors [11–13].

A multimodal approach using anesthetic agents and regional analgesics enhances pain relief and minimizes opioid side effects. Non-opioid analgesics, such as propacetamol and nefopam, are preferred due to their effectiveness and safety profiles. Propacetamol is ideal for kidney surgeries due to its safety in patients with kidney dysfunction [14–16]. Nefopam, a centrally acting analgesic, reduces opioid use and associated drowsiness without causing platelet dysfunction, making it suitable for kidney transplant patients [17, 18].

This study evaluates the effects of nefopam and propacetamol on opioid dose, pain, and quality of recovery in living kidney donors undergoing rectus sheath block after HALDN.

## Methods

### Ethical considerations

**Fig. 1:**
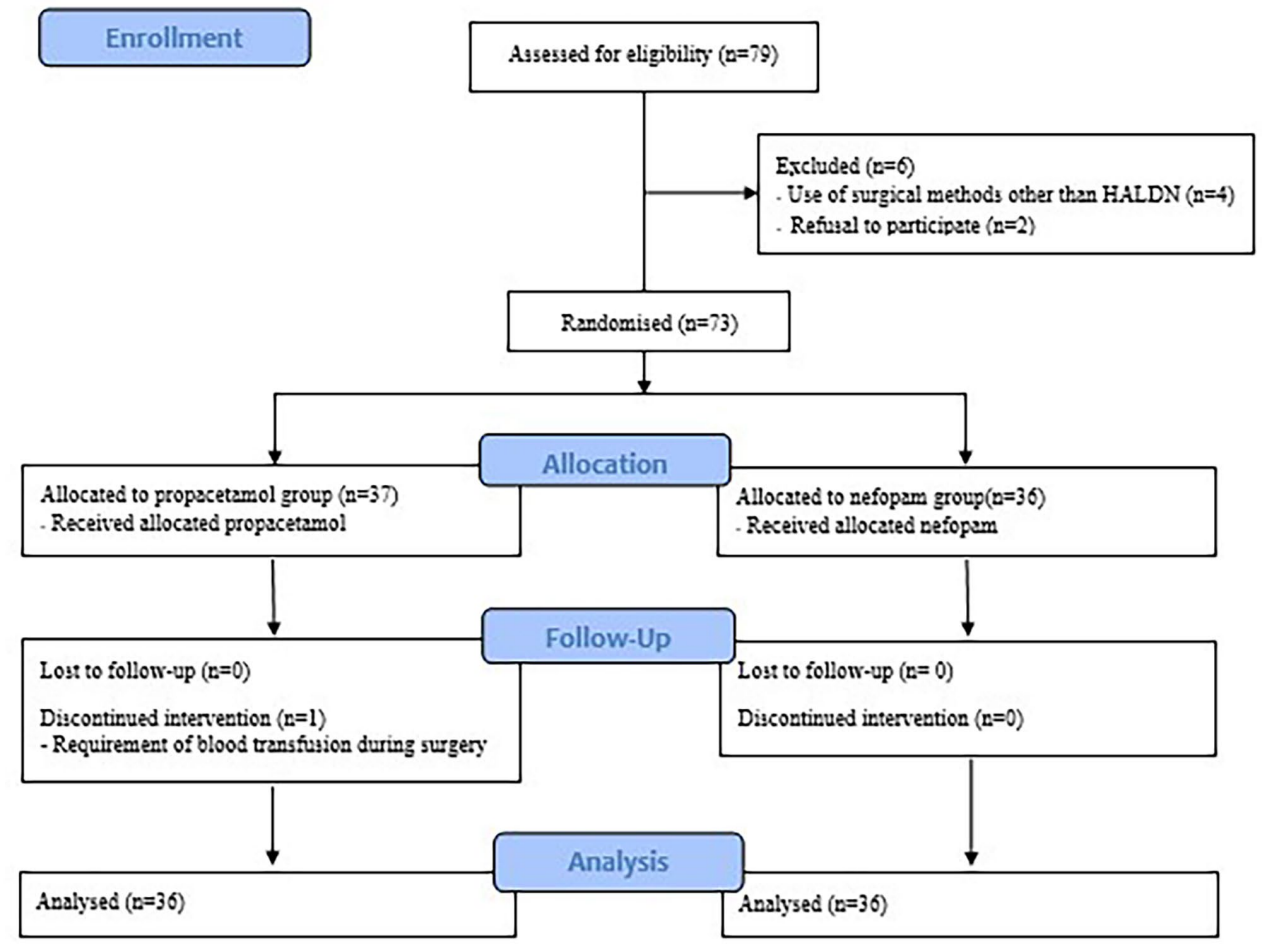
Consolidated standards of reporting trials flow diagram of participant enrollment and study process

The prospective, randomized controlled trial was conducted at Seoul St. Mary’s Hospital, Seoul, Korea. The study protocol was approved by the Institutional Review Board and Ethics Committee of Seoul St. Mary’s Hospital on 02/03/2022 (approval no.: KC22OISI0056). The study was conducted in accordance with the Declaration of Helsinki. The trial was registered prior to patient enrollment in the clinical trial database using the Clinical Research Information Service (registration no.: KCT0007351, Date of registration: 03/06/2022). Written informed consent was obtained from each participant on the day before surgery; the study was conducted between 05/06/2022 and 10/11/2022. The study was performed in accordance with the Consolidated Standards of Reporting Trials guidelines (Fig. 1).

### Study population

The study included healthy donors aged 19–75 years with an American Society of Anesthesiologists (ASA) physical status I or II who were scheduled for elective HALDN. We excluded individuals who refused to participate and individuals with ASA classification III or higher, a history of surgical procedures other than HALDN, emergency operations, reoperations, or conversion to open surgery. Additional exclusion criteria related to the rectus sheath block included allergies to ropivacaine, local skin infections at the nerve block site, significant pain or painful diseases, mental illness, alcoholism, or long-term use of analgesic or anticoagulation medication (continuous use for more than 3 months). Individuals with intraoperative hemodynamic instability due to significant bleeding and those requiring blood transfusion were also excluded.

Eligible healthy living donors were randomly assigned to either the nefopam group or the propacetamol group for comparison.

### Randomization and blinding

Living donors were randomized to the propacetamol or nefopam group using a web-based generator for stratified block randomization (www.random.org). Research staff opened sequentially numbered, opaque envelopes for each donor to determine their group assignment. This random allocation was performed in the surgery waiting room and conveyed to the drug preparation room in a sealed envelope. Drugs were prepared based on the assigned numbers by an anesthesia nurse who was not involved in outcome assessment. The prepared propacetamol and nefopam, made indistinguishable, were delivered to the operating room. To maintain objectivity, all anesthesiologists and healthcare providers assessing postoperative outcomes were unaware of the group assignments.

### Interventions during surgery

Propacetamol was prepared by mixing 2 g (1 g/vial of Denogan × 2 vials; Yungjin Pharm., Seoul, Republic of Korea) with 100 mL of 0.9% normal saline [19]. Similarly, nefopam was prepared by combining 40 mg (20 mg/2 mL nefopam hydrochloride × 2 vials; Myungmoon Pharm., Seoul, South Korea) with 100 mL of 0.9% normal saline [20]. Each patient received a single dose of one of these solutions, with 30 min of slow intravenous administration that ended immediately before skin closure, to evaluate postoperative analgesic efficacy.

### Surgery and anesthesia

HALDN was performed by an experienced surgeon using a previously described surgical technique [21]. Briefly, living donors were positioned in a partial lateral decubitus position with the table flexed to extend the flank. After the surgical site had been cleaned with povidone-iodine, a 7-cm supraumbilical incision was made to insert the hand-assistance device. A surgeon’s hand, working port, and 10-mm 30° laparoscope were inserted into the abdomen. Pneumoperitoneum was established, followed by the insertion of two additional ports for laparoscopic tools. The procedure comprised medial reflection of the colon, dissection of Gerota’s fascia from the kidney, and sharp dissection of kidney attachments, sparing the renal hilum. The gonadal, lumbar, and adrenal veins were ligated, and ureteral dissection was performed. After the renal artery and vein had been freed, mannitol (30 g) was administered. The ureter was divided at the iliac vessels, the renal artery was clipped and incised, and the renal vein was stapled. The kidney was removed through the abdominal incision.

During anesthesia, blood pressure, heart rate, electrocardiography, oxygen saturation, bispectral index, and train-of-four were routinely monitored. Anesthesia was induced using 2 mg/kg propofol and confirmed using train-of-four; 0.6 mg/kg rocuronium was administered for endotracheal intubation. Mechanical ventilation was adjusted to maintain optimal respiratory parameters. Anesthesia was maintained with 2% propofol and 2 mg remifentanil, using effect site control and Minto’s model, respectively, to ensure a bispectral index of 40–60 and systolic blood pressure < 20% from baseline. Antiemetics (5 mg dexamethasone, 75 μg palonosetron at the start of anesthesia, and 0.3 mg ramosetron at the end of anesthesia) were administered to reduce postoperative nausea and vomiting.

### Rectus sheath block (RSB) procedure

**Fig. 2:**
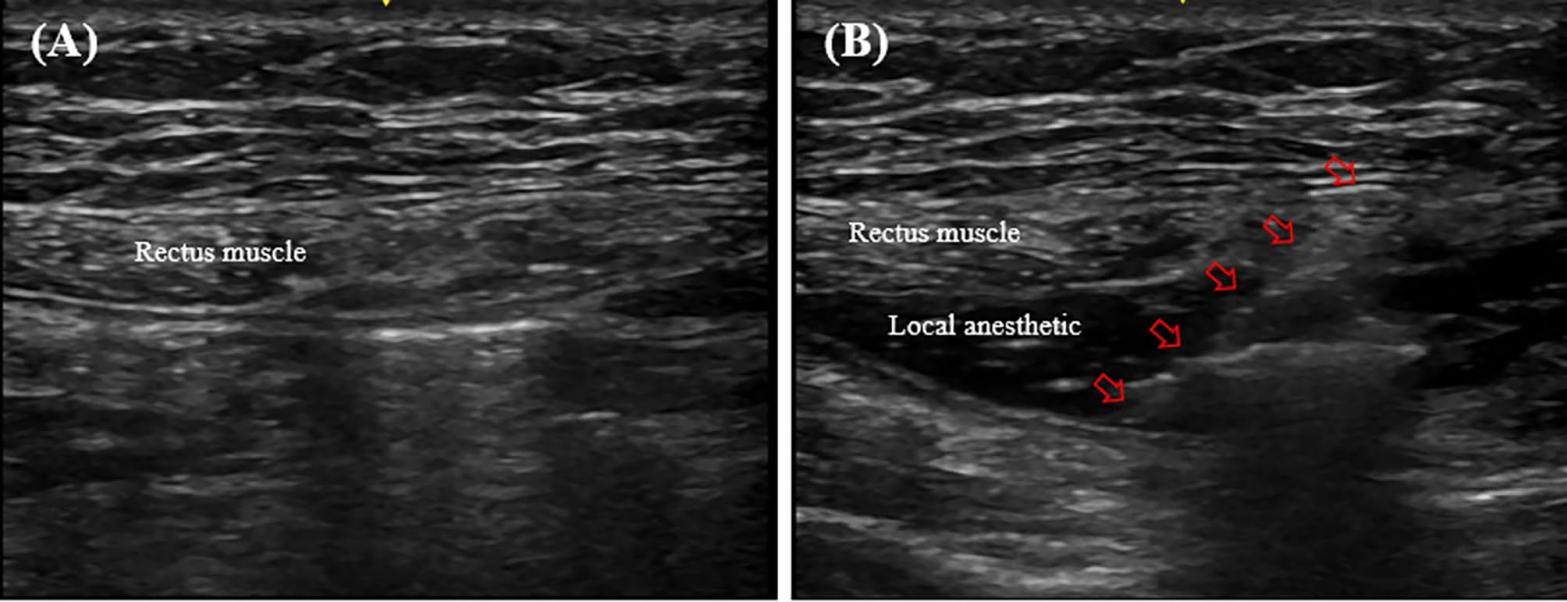
Performance of the rectus sheath block. (**A**) Anatomy of the rectus muscle, and (**B**) Application of the block. The arrows indicate the path of the needle

Immediately after the induction of general anesthesia, RSB was administered by a single experienced attending anesthesiologist who was not involved in the study (Fig. 2). An ultrasound probe was placed transversely above the umbilicus on the rectus abdominis muscle. Under real-time ultrasound guidance, a 22-G Tuohy-type epidural needle was carefully advanced in-plane using a mediolateral approach to reach the apex between the muscle and the posterior sheath, avoiding nearby vessels. After confirming the absence of blood return, 20 mL of 0.375% ropivacaine (prepared by mixing 10 mL of 0.75% ropivacaine with 10 mL of normal saline) was administered on one side of the rectus muscle. This procedure was then repeated on the opposite side, resulting in a total administration of 40 mL of 0.375% ropivacaine.

### Opioid-based pain control

The study used an intravenous patient-controlled analgesia (PCA) device (AutoMed 3200; Ace Medical, Seoul, South Korea), loaded with 1,000 μg fentanyl and 0.3 mg ramosetron in 100 mL of normal saline. The PCA device was programmed to administer a 0.5-mL basal infusion and a 2-mL bolus with a 10-minute lockout period. Donors were provided with PCA analgesia at all times to ensure continuous pain management postoperatively.

We assessed Numeric Rating Scale (NRS) scores at regular intervals, including at rest and during coughing, at 1, 6, 12, and 24 h postoperatively. For participants with an NRS score of ≥ 6, additional intravenous fentanyl doses were administered as needed. This threshold was chosen based on established clinical practices, where an NRS score of 6 or above indicates moderate to severe pain, necessitating prompt and effective analgesic intervention [22]. Approval for additional doses was obtained from attending physicians and nurses who were not involved in patient care or data collection, ensuring unbiased and consistent pain management.

### Pain outcomes

The primary outcome of the study was the total PCA volume used on postoperative day (POD) 1. Secondary outcomes included postoperative pain as determined by the NRS, with scores ranging from 0 (no pain) to 10 (severe pain), at 1 h postoperatively in the post-anesthesia care unit (PACU) and on POD 1 in the ward. Pain was evaluated at the umbilical site (representing parietal pain from the skin incision) and the flank area (representing visceral pain from the kidney graft and surrounding tissues) at rest and during coughing [7, 23]. Additionally, the total quantity of rescue fentanyl administered to each donor within the 24-h postoperative period was recorded.

### Quality of recovery

The Korean adaptation of the Quality of Recovery-15 (QoR-15 K) questionnaire was used to evaluate the quality of postoperative functional recovery on POD 1. Scores for each dimension of the questionnaire were obtained as a sum of the scores of individual items, as follows: physical comfort, items 1–4 and 13; physical independence, items 5 and 8; psychological support, items 6 and 7; emotional state, items 9, 10, 14, and 15; and pain, items 11 and 12 [24].

### Complications

On POD 1, complications related to anesthesia (e.g., nausea, vomiting, and shivering) and study drugs, such as dizziness, sweating, and tachycardia, were recorded.

### Clinical variables

Preoperative variables included sex, age, body mass index, hemoglobin, creatinine, and estimated glomerular filtration rate. Intraoperative variables included anesthesia time, propofol and remifentanil doses, hourly crystalloid infusion, hourly urine output, hypotensive events, and inotropic use.

### Sample size and statistical analysis

A preliminary study was conducted on 100 healthy living donors who received RSB from January 1, 2021, to December 31, 2021, to evaluate how much nefopam and propacetamol could reduce opioid-based PCA volumes. During this period, the mean 24-h postoperative PCA volumes were 70.2 mL for the propacetamol group and 51.8 mL for the nefopam group. To achieve 80% statistical power with a type I error rate of 5% and a standard deviation of 27.8 mL, at least 36 living donors were needed in each group. Considering an estimated 10% dropout rate, we aimed to enroll 79 living donors to ensure the robustness and reliability of our results.

The Shapiro-Wilk test was used to determine data distribution normality. Normally distributed data were compared using unpaired *t*-tests, whereas non-normally distributed data were compared using the Mann-Whitney *U* test. Categorical data were compared using Pearson’s *χ*^2^ test or Fisher’s exact test. Data are presented as means ± standard deviations or number (%), as appropriate. *P* values < 0.05 were considered indicative of statistical significance. Statistical analyses were performed using SPSS for Windows (ver. 24.0; IBM Corp., Armonk, NY, USA).

## Results

Of the 79 eligible donors, seven were excluded due to the use of surgical procedures other than HALDN (*n* = 4), refusal to participate (*n* = 2), or a need for intraoperative blood transfusion (*n* = 1). In total, 72 donors were assigned to the propacetamol (*n* = 32) and nefopam (*n* = 32) groups (Fig. 1).

### Demographic variables

This study involved 72 living donors with a mean age of 49.7 ± 12.9 years; 43 (59.7%) participants were women. The study groups had similar preoperative and intraoperative characteristics (Table 1).

**Table 1:** Comparison of preoperative and intraoperative variables between the two groups

Group	Propacetamol group	Nefopam group	*P* value
	(*n* = 36)	(*n* = 36)	
**Preoperative variables**			
Demographics			
Sex (Male / Female)	18 (50%) / 18 (50%)	11 (30.6%) / 25 (69.4%)	0.18
Age (years)	48 ± 13.9	52 ± 11.6	0.19
BMI (kg/m^2^)	24.5 ± 2.8	24.0 ± 2.8	0.4
Laboratory findings			
Hb (g/dL)	14.4 ± 1.6	14.0 ± 1.3	0.26
Creatinine (mg/dl)	0.8 ± 0.2	0.7 ± 0.1	0.25
eGFR (mL/min/1.73m^2^)	101.2 ± 22.6	131.1 ± 44.6	0.5
***Intraoperative variables***			
Anesthesia time (min)	121.2 ± 59.3	212 ± 70.6	0.24
Infusion drug dose			
Propofol (mg)	997.7 ± 326.5	1072.8 ± 412.4	0.4
Remifentanil (mcg)	862.5 ± 476.8	1012.4 ± 434.1	0.17
Hourly crystalloid infusion (ml/kg/hr)	5.6 ± 1.9	5.1 ± 1.8	0.32
Hourly urine output (ml/kg/hr)	2.0 ± 1.9	1.8 ± 1.6	0.76
*Hypotensive events and drugs*			
Hypotension (frequency of occurrence)	5 (13.9%)	2 (5.6%)	0.26
Ephedrine (number of ephedrine)	1 (2.8%)	5 (13.9%)	0.08

Abbreviations: BMI: body mass index; Hb: Haemoglobin; eGFR: expected glomerular filtration rate

Values are expressed as mean ± SD and number (proportion)

### Pain

**Fig. 3:**
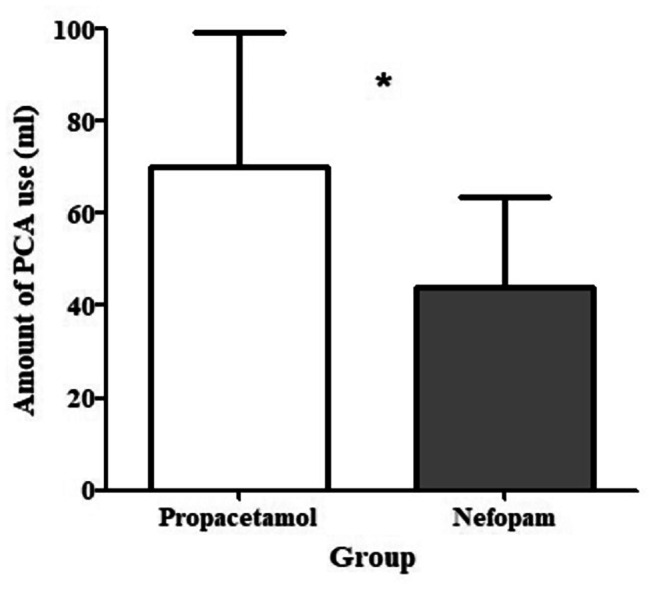
Comparison of patient-controlled anesthesia usage on postoperative day 1 between the two groups

The mean dose of intravenous PCA was significantly lower in the nefopam group (44.5 ± 19.3 mL) than in the propacetamol group (70.2 ± 29.0 mL; *p* < 0.001; Fig. 3).

**Fig. 4:**
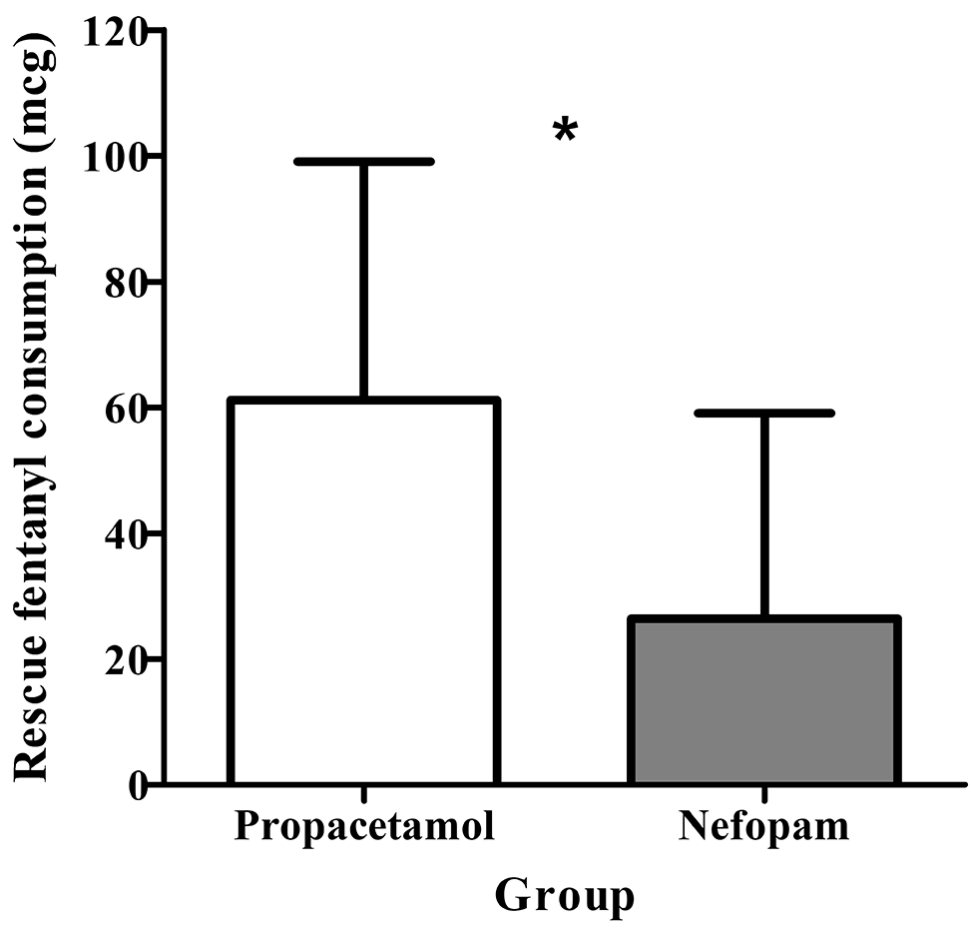
Comparison of rescue dose of fentanyl in the post-anesthesia care unit between the two groups

In the PACU, pain scores for both flank and umbilical pain at rest and during coughing were lower in the nefopam group than in the propacetamol group (Table 2). Additionally, the mean rescue dose of fentanyl was lower in the nefopam group (44.1 ± 19.3 μg) than in the propacetamol group (70.2 ± 29.0 μg; *p* < 0.001; Fig. 4).

**Table 2:** Comparison of postoperative pain scores between the two groups

Group	Propacetamol group	Nefopam group	*P* value
	(*n* = 36)	(*n* = 36)	
*Pain score (NRS) in the PACU*			
Flank pain at rest	3.4 ± 2.0	1.4 ± 0.8	< 0.001
Flank pain during cough	6.3 ± 1.9	3.8 ± 1.4	< 0.001
Umbilical pain at rest	2.8 ± 2.5	1.1 ± 0.5	< 0.001
Umbilical pain during cough	4.4 ± 2.6	2.3 ± 1.0	< 0.001
*Pain score (NRS) in the ward*			
Flank pain at rest	3.8 ± 2.7	3.7 ± 2.5	0.93
Flank pain during cough	6.2 ± 2.5	5.8 ± 2.4	0.47
Umbilical pain at rest	4.2 ± 2.8	3.3 ± 2.1	0.12
Umbilical pain during cough	6.8 ± 2.7	5.7 ± 2.2	0.07

Abbreviations: NRS: Numeric Rating Scale; PACU: post-anesthesia care unit; PONV: postoperative nausea and vomiting

Values are expressed as mean ± SD and number (proportion)

During the 24-h postoperative period in the ward, pain scores for flank and umbilical areas both at rest and during coughing were similar between the two groups (Table 2). Furthermore, the rescue doses of fentanyl were similar between the propacetamol and nefopam groups (29.2 ± 25.0 and 25.0 ± 25.4 μg, respectively; *p* = 0.485).

### QoR-15 K

**Fig. 5:**
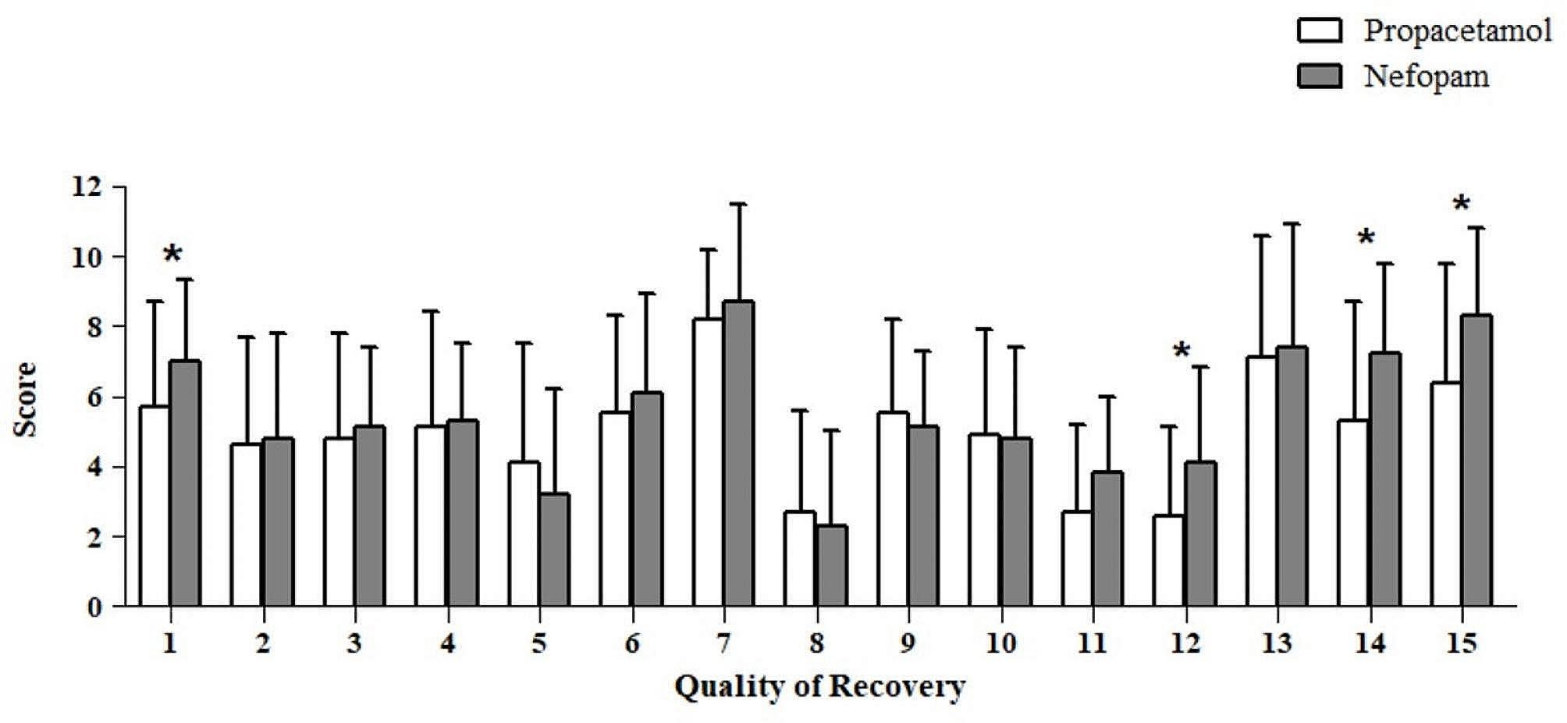
Comparison of global and subdimension scores from the quality of Recovery-15 questionnaire on postoperative day 1 between the two groups

Global QoR-15 K scores were comparable between the two groups. However, subgroup analysis revealed significant differences in certain subdimensions, such as breathing, pain severity, and the levels of anxiety and depression. Respiratory distress, severe pain, anxiety, and depression were less common in the nefopam group than in the propacetamol group (Fig. 5; Table 3).

**Table 3:** Comparison of global and subdimension scores from the quality of Recovery-15 questionnaire on postoperative day 1 between the two groups

Group	Propacetamol group (*n* = 36)	Nefopam group (*n* = 36)	*P* value
**Total QoR-15 K score**	74.9 ± 26.4	82.6 ± 19.2	0.9
1 Able to breathe easy	5.7 ± 3.0	7.0 ± 2.3	0.04
2 Been able to enjoy food	4.6 ± 3.1	4.8 ± 3.0	0.8
3 Feeling rested	4.8 ± 3.0	5.1 ± 2.3	0.7
4 Have had a good sleep	5.1 ± 3.3	5.3 ± 2.2	0.8
5 Able to look after personal toilet and hygiene unaided	4.1 ± 3.4	3.2 ± 3.0	0.2
6 Able to communicate with family or friends	5.5 ± 2.8	6.1 ± 2.8	0.3
7 Getting support from hospital doctors and nurses	8.2 ± 2.0	8.7 ± 1.8	0.3
8 Able to return to work or usual home activities	2.7 ± 2.9	2.3 ± 2.7	0.5
9 Feeling comfortable and in control	5.5 ± 2.7	5.1 ± 2.2	0.4
10 Having a feeling of general well-being	4.9 ± 3.0	4.8 ± 2.6	0.8
11 Moderate pain	2.7 ± 2.5	3.8 ± 2.2	0.07
12 Severe pain	2.6 ± 2.5	4.1 ± 2.7	0.018
13 Nausea or vomiting	7.1 ± 3.5	7.4 ± 3.5	0.7
14 Feeling worried or anxious	5.3 ± 3.4	7.2 ± 2.6	0.008
15 Feeling sad or depressed	6.4 ± 3.4	8.3 ± 2.5	0.011

Abbreviations: QoR: quality of recovery

Values are expressed as mean ± SD

### Postoperative complications

The incidences of postoperative complications were similar between groups in the PACU and ward; none of the participants experienced sweating or tachycardia (Table 4).

**Table 4:** Comparison of postoperative complications between the two groups

Group	Propacetamol group	Nefopam group	*P* value
	(*n* = 36)	(*n* = 36)	
*In the PACU*			
PONV	1 (2.8%)	1 (2.8%)	> 0.999
Dizziness	0	0	> 0.999
Shivering	3 (8.3%)	0	0.24
Sweating / Tachycardia	0	0	> 0.999
*In the ward*			
PONV	15 (41.7%)	11 (30.6%)	0.46
Dizziness	4 (11.1%)	4 (11.1%)	> 0.999
Shivering	0	0	> 0.999
Sweating / Tachycardia	0	0	> 0.999

Abbreviations: NRS: Numeric Rating Scale; PACU: post-anesthesia care unit; PONV: postoperative nausea and vomiting

Values are expressed as mean ± SD and number (proportion)

## Discussion

Our results suggest that nefopam is more effective than propacetamol in terms of minimizing total opioid consumption on POD 1 after HALDN. In the PACU, donors who received nefopam at the end of surgery experienced a significant decrease in visceral pain and an increase in RSB effectiveness for parietal pain alleviation, both at rest and during coughing. The improved pain in the early postoperative period may positively influence self-reported metrics related to the quality of postoperative recovery, such as ease of breathing; intensity of severe pain; and feelings of worry, anxiety, sadness, and depression.

Opioid medications are essential for severe postoperative pain management but pose risks of dependency and abuse [25]. Healthy living donors undergoing major surgeries are particularly vulnerable to opioid-related complications. To mitigate these risks, multimodal pain management regimens, including regional anesthesia (e.g., nerve blocks), non-opioid pain medications (such as acetaminophen, nefopam, and NSAIDs), adjunctive therapies (gabapentinoids, muscle relaxants), and non-pharmacological approaches (physical therapy, psychological support), are increasingly used [26]. Intraoperative RSB at our institution effectively manages pain from skin incisions without the adverse effects of epidural and spinal analgesia, though it has limited impact on visceral pain [27]. Propacetamol and nefopam are preferred for visceral pain due to their low nephrotoxicity and safety for patients with renal impairment, reducing opioid use and associated side effects [14, 17].

The OCTOPUS study examined the impact of paracetamol, nefopam, ketoprofen, morphine, and their combinations on postoperative morphine use and pain relief. The combination of these three non-opioid drugs significantly reduced morphine use and improved pain relief up to 48 h postoperatively compared to single-drug use or the control group. Paracetamol at 4 g/day had similar analgesic efficacy to nefopam at 80 mg/day [28]. Our study found nefopam more effective than propacetamol in relieving postoperative visceral pain and enhancing RSB effectiveness for parietal pain in healthy living donors, particularly those with renal impairment who must avoid NSAIDs [29]. Nefopam reduced the morphine requirement by 30–50%, with a 20 mg dose comparable to 12 mg of morphine in effectiveness [18, 30]. In older patients with lumbar spinal stenosis, nefopam reduced dysesthesia and improved patient satisfaction at 12 and 24 h postoperatively compared to controls [31]. For patients undergoing hepatic resection, nefopam lowered resting pain and morphine use on POD 1 compared to propacetamol, though cough-induced pain was similar between the two drugs [32]. Nefopam also improved postoperative well-being, reduced pain, and enhanced patient comfort as indicated by QoR-15 K scores while reducing opioid requirements. Delayed onset of analgesia from regional anesthesia blocks can lead to inadequate pain relief within the first postoperative hour, correlating with poorer overall recovery and higher opioid needs [33].

Nefopam is generally well-tolerated but can cause sweating, nausea, tachycardia, malaise, and vomiting postoperatively. While sweating is common, it is usually not serious. Tachycardia can be severe in patients with limited cardiac function [34]. In our study, living donors did not experience sweating or tachycardia, and incidences of postoperative nausea, vomiting, and dizziness were similar between nefopam and propacetamol groups. Side effects requiring discontinuation are rare with nefopam when administered in appropriate doses. However, life-threatening events have been reported in overdoses, so careful monitoring is essential [34].

This study had some limitations. First, we selected a healthy donor population, which may not reflect outcomes among patients with poor health; our focus on this population may limit the generalizability of the findings. Second, we did not compare efficacy between nefopam and propacetamol, leading to difficulty in accurately determining their equivalent doses in relation to morphine. Third, although RSB was effective for managing parietal pain, our results may not be applicable to other block techniques. Additionally, the use of an epidural needle for the RSB, rather than specialized plane block needles, may affect the precision and safety of the block. Despite these limitations, our study rigorously evaluated the efficacy of nefopam as a component of a multimodal pain management strategy, particularly for parietal pain relief via RSB. Future studies should focus on comparing effective doses of non-opioid drugs that provide opioid-sparing results, especially within the context of parietal block-based approaches, as recommended by the Enhanced Recovery After Surgery (ERAS) protocol [16, 26].

## Conclusions

Nefopam, an effective non-opioid analgesic for surgical patients, has not been extensively evaluated, particularly in studies differentiating between visceral and parietal pain. This gap is primarily due to the lack of focus on regional block techniques in prior research. Given the increasing importance of regional blocks, evaluating the efficacy and dosage of relevant drugs is essential. Our study highlighted nefopam’s effectiveness in relieving visceral pain and enhancing block effects, demonstrating its reliability and minimal adverse effects compared to propacetamol. Nefopam can be a key component of analgesic strategies in the ERAS protocol for living donors. Further research is required to determine the optimal dosages and potential synergistic effects of other non-opioid drugs. Additionally, comparative studies between the rectus sheath block and newer abdominal plane blocks, as well as their combinations, are essential to achieve the goal of minimizing opioid exposure in healthy living donors.

## Data Availability

Data is provided within the manuscript or supplementary information files.

## Abbreviations

HALDN: Hand-assisted laparoscopic donor nephrectomy
NRS: Numeric rating scale
PACU: Post-anesthesia care unit
PCA: Intravenous patient-controlled analgesia
POD: Postoperative day
QoR-15K: Korean adaptation of the Quality of Recovery-15 questionnaire
RSR: Rectus sheath block
